# The effect of acute exercise on environmentally induced symptoms of dry eye

**DOI:** 10.14814/phy2.14262

**Published:** 2020-01-29

**Authors:** Daniel J. Peart, Ian H. Walshe, Emma L. Sweeney, Emily James, Thomas Henderson, Alasdair F. O'Doherty, Alison M. McDermott

**Affiliations:** ^1^ Department of Sport, Exercise and Rehabilitation Northumbria University Newcastle‐upon‐Tyne United Kingdom; ^2^ Department of Applied Sciences Northumbria University Newcastle‐upon‐Tyne United Kingdom

**Keywords:** Dry eye, exercise, MMP‐9, Schirmer, tears

## Abstract

The purpose of this study was to investigate the effects of acute exercise on environmentally induced symptoms of dry eye. Twelve participants without dry eye disease volunteered to complete three experimental visits in a randomized order; (1) control condition seated for 1 h at a relative humidity (RH) of 40% (CONT), (2) dry condition seated for 1 h at a RH of 20% (DRY), and (3) exercise condition seated for 40 min followed by 20 min of cycling exercise at a RH of 20% (EXER). Tear volume, tear matrix metalloproteinase 9 (MMP‐9), perception of dry eye symptoms (frequency and severity), core temperature, and ocular surface temperature (OST) were measured at the end of each exposure. The perception of dry eye frequency and MMP‐9 concentration were significantly higher in DRY compared to CONT (*P* < 0.012), with no differences in EXER compared to CONT. The results suggest that an acute bout of exercise may attenuate symptoms of environmentally induced dry eye, and warrant further research.

## Graphical abstract

Exposure to a desiccating environment increased the perception of dry eye symptoms and concentration of matrix metalloproteinase 9 (MMP‐9) in tears compared to a control environment. These findings were absent when participants completed an acute bout of exercise in the desiccating environment, suggesting that exercise may attenuate symptoms.

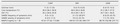

## Introduction

Chronic dryness of the ocular surface can manifest as dry eye disease, a condition characterized by discomfort, inflammation, and blurring of vision between blinks (Bron et al. 2017). These symptoms can present acutely or chronically (Gayton 2009; Schaumberg et al. 2009) and the latter may lead to damage to the ocular surface. Acute presentation of dry eye has been observed in people working (Uchiyama et al. 2007; Sano et al. 2018) and exercising (Gayton 2009; Qiao 2012) in desiccating environments, in contact lens wearers (Nichols and Sinnott 2006), and in people working in irregular shift patterns (Makateb and Torabifard 2017). One hour of exposure to a relative humidity (RH) of 5% can induce dry eye in otherwise non‐sufferers (Abusharha and Pearce 2013). The authors proposed that the thinner tear lipid layer observed in 5% RH may be influenced by alterations in protein structures on the corneal surface (Abusharha and Pearce 2013). Matrix metalloproteinase 9 (MMP‐9) is an endopeptidase involved in extracellular matrix remodeling and inflammation after corneal surface damage and has become a key biological marker for dry eye due to its direct relationship with dry eye severity (Kaufman 2013). It is hypothesized that MMP‐9 disrupts the corneal epithelial barrier facilitating ulceration and disrupting wound healing (Kaufman 2013). Therefore, acute exposure of non‐dry eye sufferers to low RH conditions may provide a useful model to investigate interventions that may influence dry eye, and MMP‐9 appears to be a key objective variable of dry eye‐induced ocular surface dysfunction.

Underexplored interventions that may influence dry eye symptoms are exercise and physical activity. In a cross‐sectional study, Kawashima et al. (2014) observed that participants without dry eye disease tended to have higher levels of physical activity as measured by the International Physical Activity Questionnaire (IPAQ). It was postulated that reduced physical activity may exacerbate dry eye by increasing the risk of chronic inflammatory diseases and oxidative stress, which can contribute to short tear break‐up time. However, other aspects of sedentary behavior may contribute to the risk of dry eye, therefore physical inactivity may not be causal. For example, increased visual display unit use can reduce blinking rate and consequently dryness of the ocular surface increases. Sano et al. (2018) recruited office workers suffering from dry eye symptoms to a 10‐week home‐based core exercise intervention, and found an improvement in subjective dry eye symptoms. Similarly, Kawashima et al. (2018) enlisted office workers for a 2‐month multidisciplinary lifestyle intervention including increased physical activity and also found improvements in subjective symptoms. These intervention studies add to the observational data reported by Kawashima et al. (2014), but objective measurements are required to determine the efficacy of exercise in attenuating dry eye symptoms. It remains unknown whether an acute bout of exercise influences symptoms of dry eye. Theoretically, exercise‐induced increases in core body temperature may increase ocular surface temperature (OST), improving the fluidity of the meibomian lipids within the tear film and inhibit evaporation from the eye (Bron et al. 2004). Conversely, the risk of dry eye could be increased by exercise because increased OST may elevate the rate of tear evaporation (Borchman et al. 2009).

The aim of this study was to investigate the effect of acute exercise on environmentally induced symptoms of dry eye.

## Methods

### Participants

Twelve (seven male, five female; mean ± SD age 26.7 ± 5.7 years, mass 72.9 ± 11.6 kg, stature 1.73 ± 0.09 m, peak power output 294.0 ± 54.8 W) non‐dry eye sufferers volunteered to take part in this study. All participants were screened using the ocular surface disease index (OSDI) survey to confirm that they did not suffer from dry eye. Participants undertook a preliminary Schirmer test to familiarize them to the procedure and ensure that they produced a reading of at least 5 mm to allow for later analysis (see procedures for full description). All participants provided written and verbal consent, and all procedures were approved by the ethics committee at Northumbria University (reference 897).

### Experimental design

Participants reported to the laboratory on four occasions: (1) preliminary measures and maximal aerobic exercise test, (2) control condition (CONT; seated for 60 min at 25°C 40% RH), (3) dry condition (DRY; seated for 60 min at 25°C 20% RH), and (4) exercise condition (EXER; seated for 40 min, 100 W warm up for 5 min, and cycle at 40% Δ (190.4 ± 35.7 W) for 15 min under dry conditions). Visits 2–4 were completed in a counterbalanced randomized order (https://www.randomizer.org/) and took place within an environmental chamber (Peak Performance Chamber Series 2009, T.I.S. Services, UK) to control the temperature and RH. The environmental conditions were modified from (Abusharha and Pearce 2013) to conditions (temperature and RH) that could be maintained within the environmental chamber during the exercise trial. The following measurements were taken at the end of each 60‐min exposure: (1) tear production, (2) core temperature, (3) OST, and (4) perceived dryness of the eye.

### Procedures

Prior to prescription of exercise, participants completed an incremental exercise test on a cycle ergometer (Velotron Pro cycle ergometer, Racermate Inc., USA) until volitional exhaustion. Participants performed a warm up of unloaded cycling (2–5 min) and progressed 30 W·min^−1^ in a ramped fashion until volitional exhaustion or until 50 RPM could not be maintained by the participant. Expired gas was collected and analyzed using an online breath‐by‐breath gas analyzer (Cortex Metalyzer 3B, Biophysik, Germany). Breath‐by‐breath data were averaged using a rolling average of the middle 5 of every 7 breaths. The ventilatory anaerobic threshold was identified using the modified V‐slope method (Beaver et al. 1986) and confirmed with ventilatory equivalents for the rate of oxygen uptake (${\dot{V}}_{E}$/$\dot{V}$O_2_) and carbon dioxide production (${\dot{V}}_{E}$/${\dot{V}\text{CO}}_{2}$) (Whipp et al. 1986). Maximum $\dot{V}$O_2_ ($\dot{V}$O_2_max) was identified as the highest oxygen consumption achieved during exercise, averaged over 30 sec (Midgley et al. 2007). The 40% Δ workload was calculated by identifying the work rate at the ventilatory anaerobic threshold (140.1 ± 26.7 W), subtracting 2/3 of the ramp rate to account for the delay in oxygen kinetics (Whipp et al. 1981), and calculating 40% of the difference between this value and the peak work rate achieved. This was to ensure that participants exercised at a similar relative intensity that could be sustained for 15 min and was within the heavy exercise intensity domain (Burnley and Jones 2007).

Tear production was quantified using 40‐mm Schirmer strips (Praxisdienst, UK) which were placed 5 mm inside the lower eye lid. The eye was kept closed for 5 min as per standard procedures (Serin et al. 2007). Once the reading had been taken, each strip was placed inside a 0.5‐mL Eppendorf tube with a hole puncture in the bottom. This was then placed in a 1.5‐mL Eppendorf tube and centrifuged at 12,000 *g* for 5 min (Posa et al. 2013). The resultant supernatant was immediately stored at −80°C for later analysis of MMP‐9 using a commercially available ELISA kit (R & D Systems). MMP‐9 was only detectable in all trials for eight participants hence *n* = 8 for this variable.

Core temperature was measured using a tympanic thermometer (Braun Thermoscan 5, Germany), and OST from the center of the eye using a thermal imaging camera (TG165 Imaging IR thermometer, FLIR Systems, USA) 8 sec after a blink (Purslow et al. 2005). Perceived dryness of the eye was determined using the symptom assessment in dry eye (SANDE) questionnaire (Amparo et al. 2015), where participants answered two questions on a 100‐mm visual analogue scale: (1) indicate how often in the last hour your eyes feel dry and/or irritated, and (2) indicate how severe you feel your symptoms of dryness and/or irritation are. Change in body mass during the EXER trial was taken to ascertain if dehydration contributed to any of the findings.

### Data analysis

An a priori sample size estimation identified that 12 participants would be suitable to identify a change of 5 mm in the Schirmer test with 80% power. This was chosen because the upper range of the variation of resting Schirmer measures across three laboratory visits in 14 healthy participants was 3 mm (Serin et al. 2007). A similar difference has been reported across stages of the menstrual cycle (Versura et al. 2007). Data were checked for normal distribution via visual inspection of box plots and Q‐Q plots. Data were analyzed using PASW Statistics 22.0 for Windows (SPSS, Inc., Chicago, IL). None of the variables were normally distributed so differences were determined using the Friedman test, and where a significant (*P* < 0.05) *Χ* ^2^ was found post‐hoc Wilcoxon signed ranks tests were applied with a Bonferroni correction (*P* < 0.017). Data are presented as the median and interquartile (IQ) range.

## Results

The median and IQ range for each variable are presented in Table 1. Tear volume measured using the Schirmer strips was not significantly different between conditions (*Χ* ^2^ = 2.591, *P* = 0.274). Similarly, core temperature (*Χ* ^2^ = 3.511, *P* = 0.173) and OST (*Χ* ^2^ = 0.304, *P* = 0.859) were comparable between conditions. There was a significant main effect between conditions for the frequency (*Χ* ^2^ = 11.73, *P* = 0.003) and severity (*Χ* ^2^ = 9.8, *P* = 0.007) of perceived dry eye symptoms. The post‐hoc threshold was not met by the severity data (*P* ≥ 0.028), but the frequency of symptoms was higher in the DRY compared to CONT and EXER conditions (*P* ≤ 0.008). The concentration of MMP‐9 was different between conditions (*Χ* ^2^ = 7.75, *P* = 0.021), specifically it was higher in DRY compared to CONT (*P* = 0.012). Percentage dehydration as indicated by change in body mass during EXER was negligible (0.27% on average). The heart rate in the final minute of the exercise trial was 173 ± 13 beats per minute.

**Table 1:** A summary of experimental findings under CONT, DRY and EXER conditions (median and IQ range)

	CONT	DRY	EXER
Schirmer (mm)	11 (5–17.25)	8 (5.75–9.25)	8 (4.75–15.5)
Core temperature (°C)	36.4 (36.2–36.9)	36.6 (36.4–36.8)	37.2 (36.7–37.5)
OST (°C)	35.2 (34.6–35.9)	35.4 (34.7–36.1)	34.7 (34.1–36.0)
SANDE frequency of symptoms (mm)	9 (5–12.5)	21 (13–28.5)*†	8 (4.25–20.75)
SANDE severity of symptoms (mm)	6 (3.5–11.5)	19 (12–25.75)	7 (1.75–19.5)
MMP‐9 (ng/mL) (*n* = 8)	67.61 (77.42–113.84)	149.53 (151.60–349.17)*	88.79 (102.98–237.71)

OST, ocular surface temperature; SANDE, symptom assessment in dry eye; MMP‐9, matrix metalloproteinase 9.

* Significantly different to CONT.

^†^ Significantly different to EXER.

## Discussion

The main findings of this study are that exposure to a desiccating environment of 20% RH did not influence tear volume measured by Schirmer strips. However, 20% RH appeared to increase the perception of the frequency of dry eye symptoms and presence of MMP‐9 in tears at rest. Interestingly, the addition of exercise in the final 20 min exposure may attenuate these symptoms.

Tear volume, as measured by Schirmer strips, was not different between trials. This is in contrast to previous literature, where tear production was reduced in resting dry conditions using similar methods (Abusharha and Pearce 2013). The disparity may be due to the modest RH (20%) used in this experiment, compared to the RH (5%) employed by Abusharha and Pearce (2013). Despite no differences in tear volume, the DRY condition elicited a greater perception of dry eye symptom frequency, and increased MMP‐9 in tears. Therefore, an arguably more ecologically valid RH used in the present study appears to initiate the symptoms of dry eye.

The perception of dry eye symptom frequency was reduced in the EXER trial compared to DRY. This supports the previous research that found increasing physical activity can reduce the perception of symptoms (Kawashima et al. 2018; Sano et al. 2018). However, there is risk of a placebo effect on perceptual responses with exercise. To account for this, we included MMP‐9 as an objective biological marker of dry eye. MMP‐9 concentrations were higher in DRY compared to CONT. Although MMP‐9 was not different between EXER and DRY, the EXER condition was also not different to CONT. Therefore, it could be viewed that exercise may play a protective role in dry environments. The mechanism for the potential protective role of exercise against dry eye is unclear. Our proposed theory of increased core temperature increasing OST remains untested as core temperature was similar between conditions. Hypothetical mechanisms could include changes in blinking rate, pro‐ and anti‐inflammatory cytokine release during exercise, or changes in intraocular pressure and blood flow. However, this is speculation as these variables were not measured. Furthermore, tympanic thermometers have been shown to vary from rectal temperature by up to 0.67°C (Ganio et al. 2009), so the originally proposed mechanism cannot be discounted.

This is the first pilot study of its kind and it is important to note the limitations. The sample size estimation was based on Schirmer test results. Therefore, our secondary findings pertaining to MMP‐9 are at risk of type 1 error. There was no difference in MMP‐9 between DRY and EXER therefore the potential benefits of exercise on reducing dry eye should be interpreted with caution. Similarly, because of the small sample size of this pilot study and MMP‐9 was a secondary outcome measure, type 2 error cannot be discounted. Another factor limiting the interpretation of the MMP‐9 results is the absence of time course data following environmental condition with or without exercise exposure. During practice trials, the conditions were set at 21°C and 10% RH; however, these conditions were difficult to maintain when participants were exercising. Increasing the RH to 20% was not strictly an issue as it made the study more ecologically valid than past research, but the increase in temperature to 25°C perhaps reduces ecological validity for some parts of the world. For this pilot trial, it was important to ensure that temperature could be matched between conditions.

We believe that these findings are interesting and merit further investigation. For MMP‐9 to be used as a primary outcome measure in future studies, it would be useful to have reproducibility data on this important biological marker. Additionally, further research is warranted to extend the data collection period beyond the time point immediately following the 1‐hour condition exposure to allow further insight into the time course of any beneficial effects. It would also assist in determining whether the changes in perceived symptoms continue after the distraction of taking part in exercise.

In conclusion, this study provides some evidence that exercise may attenuate acute perceptions of dry eye symptoms and MMP‐9 in tears when exposed to a desiccating environment. Limitations to this pilot work have been acknowledged and a larger scale study is warranted before any firm conclusions are drawn. However, perhaps as important is that the results provide no evidence that exercise exacerbates acute symptoms of dry eye, although it is acknowledged that the exercise stimulus was relatively low.

## Conflict of Interest

None declared.
